# Supplementary material for: Differentiation granules, a dynamic regulator of T. brucei development
Source: Nat Commun. 2024 Apr 6;15:2972. doi: 10.1038/s41467-024-47309-1 (PMC10998879; doi:10.1038/s41467-024-47309-1)
Supplement: Supplementary file 1 — Supplementary Information [file 41467_2024_47309_MOESM1_ESM.pdf]

Supplementary figure 1

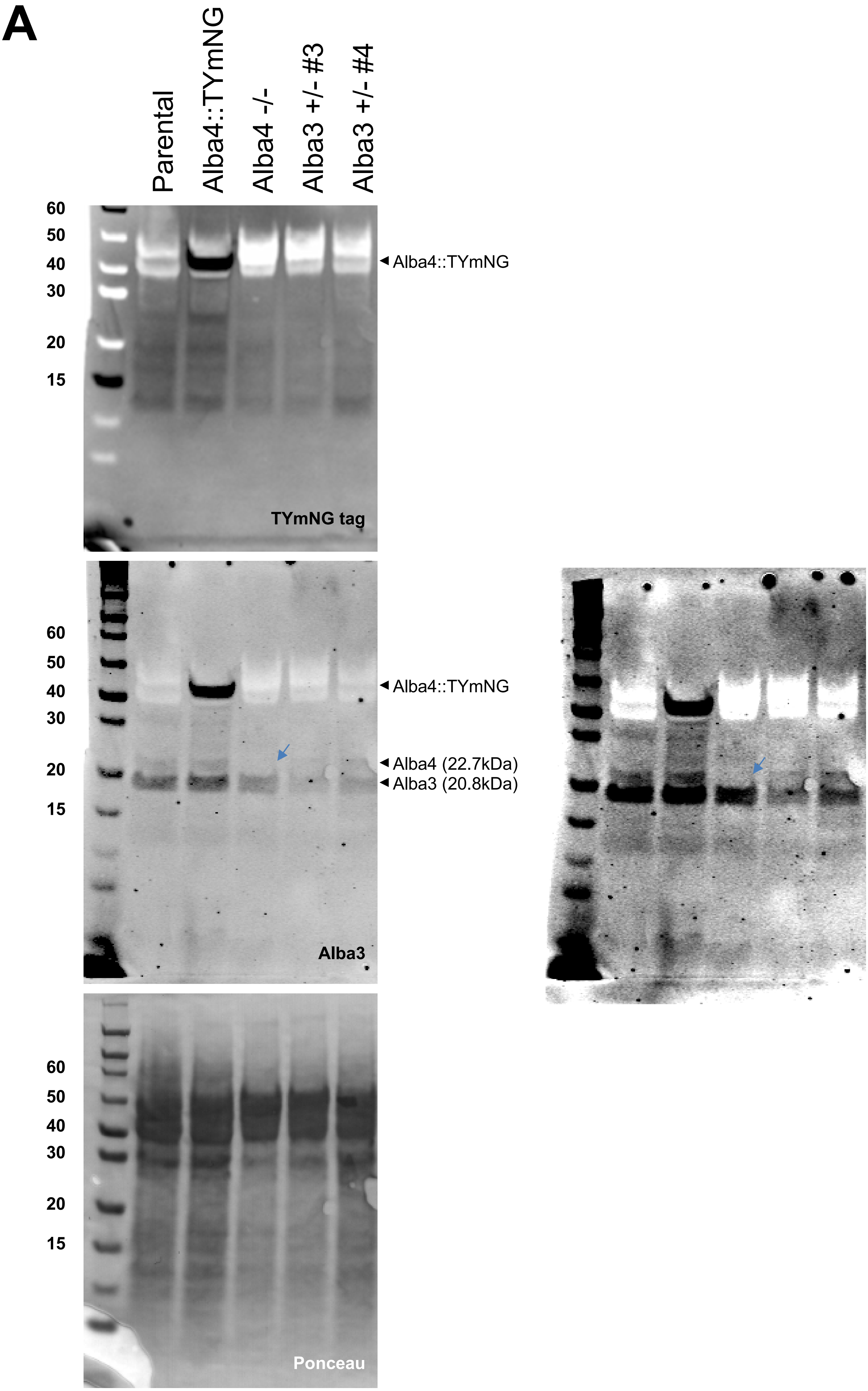

Supplementary figure 1 legend.

Western blot analysis indicates that anti-Alba 3 antibody also detects Alba 4. Cell Line: Parental = AnTat1.1 J1339, Alba 4::TYmNG = AnTat1.1 J1339 Alba 4::TYmNG, Alba 4-/- = AnTat1.1 J1339 Alba 4-/-, Alba 3+/- #3/4 = AnTat1.1 J1339 Alba 3+/- clone 3 or 4. Western blot revealed using anti-Ty tag (top panel) and anti-Alba 3 (middle panel) antibodies, and loading is controlled using ponceau staining of the membrane (lower panel). Black arrowheads indicate the expected sizes of Alba 3, Alba 4 and Alba 4::TYmNG, and the blue arrow is highlighting the missing band in the Alba 4 null cell line. A longer exposure of the blot detecting Alba 3 is shown to the right, with the missing Alba 4 cross-reacting band highlighted in the Alba 4-/- line. n=1.

Supplementary figure 2

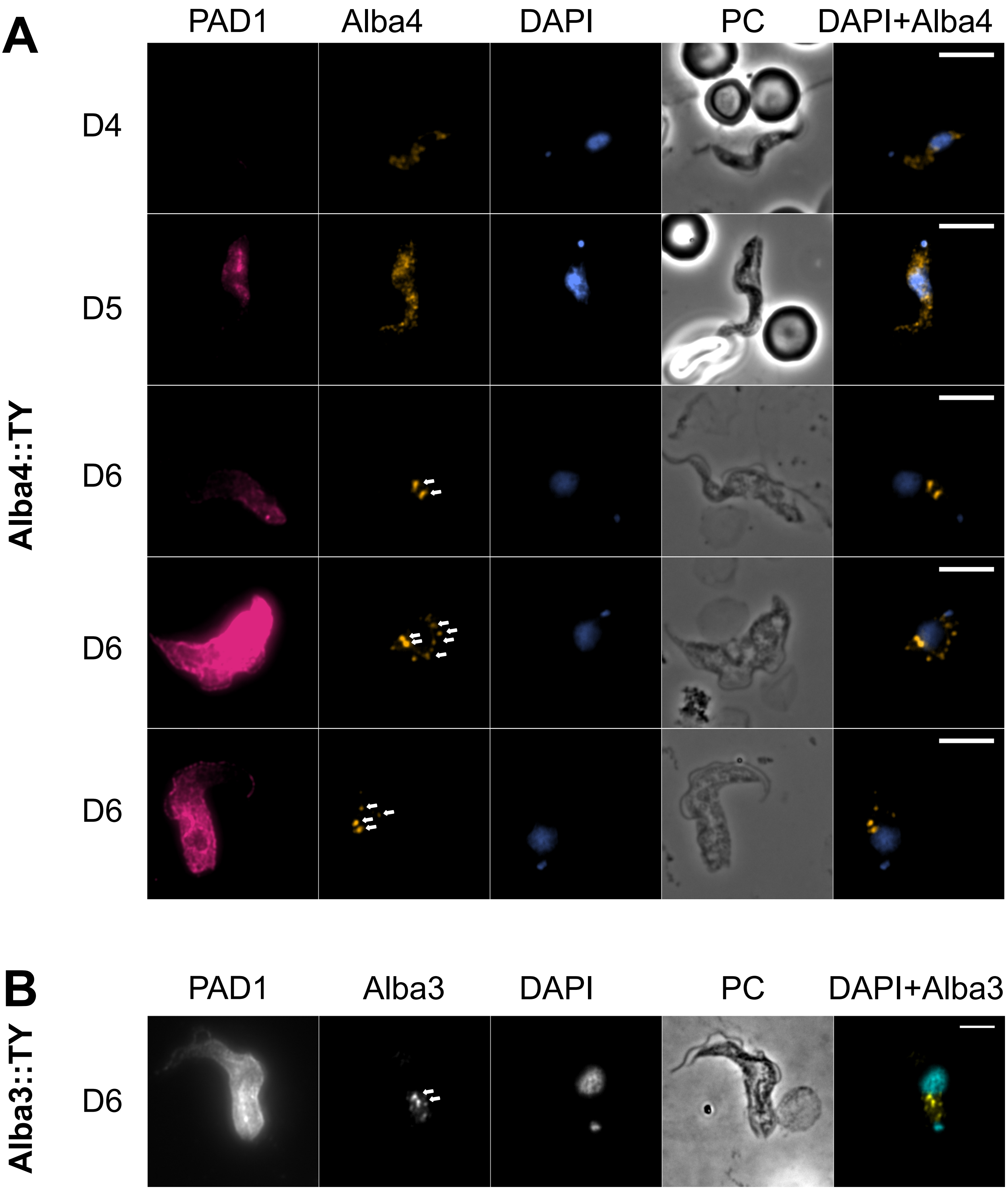

Supplementary figure 2 legend.

Alba 4 is targeted to differentiation granules that change of localisation during quorum-sensing driven differentiation. A) Immunofluorescence of blood-smears of mice infected with Alba 4::TYmNG tagged cell line from day 4 (D4) to day 6 (D6) post-infection are shown. PAD1 expression is detected using anti-PAD1 antibody (PAD1, false coloured Pink), Alba 4 localisation is revealed by anti-Ty antibody (Alba 4, false coloured Yellow), nucleus and kinetoplast using DAPI (false coloured Blue). PC Phase contrast. Acquisition settings were kept constant to detect weak positive cells, leading to saturation of the PAD1 signal on late time points. Scale bar = 10µm. Arrows highlight differentiation granules. B) Position of the Ty-mNG tag at the N- or C-terminal end of Alba 3 does not affect the protein localisation. The same experiment as in Figure 3 was performed using a cell line where Alba 3 was endogenously tagged at its C-terminal end and its granular localisation was observed during the time course of in vivo differentiation. A representative image at D6 post infection is provided.

Supplementary figure 3

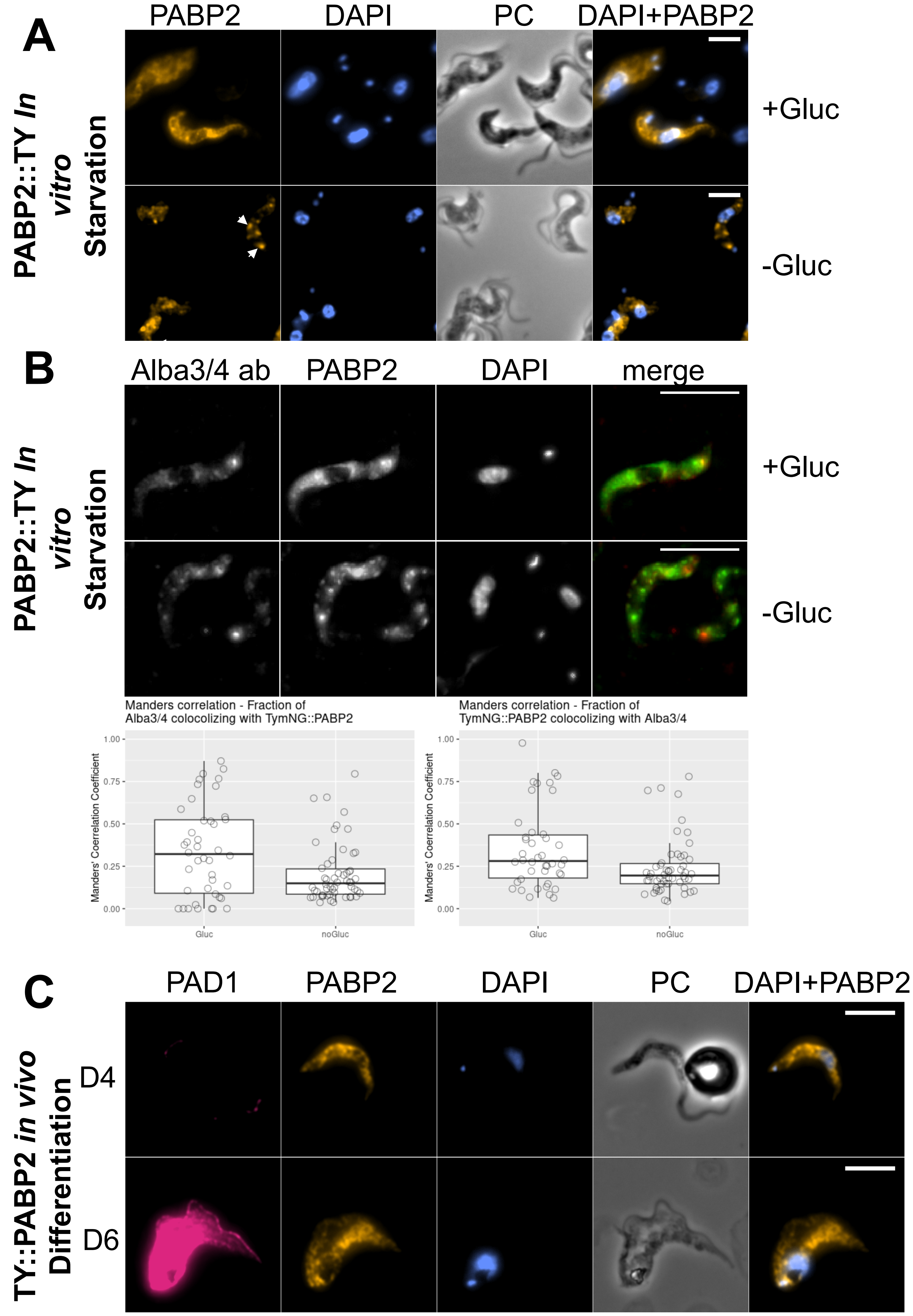

Supplementary figure 3

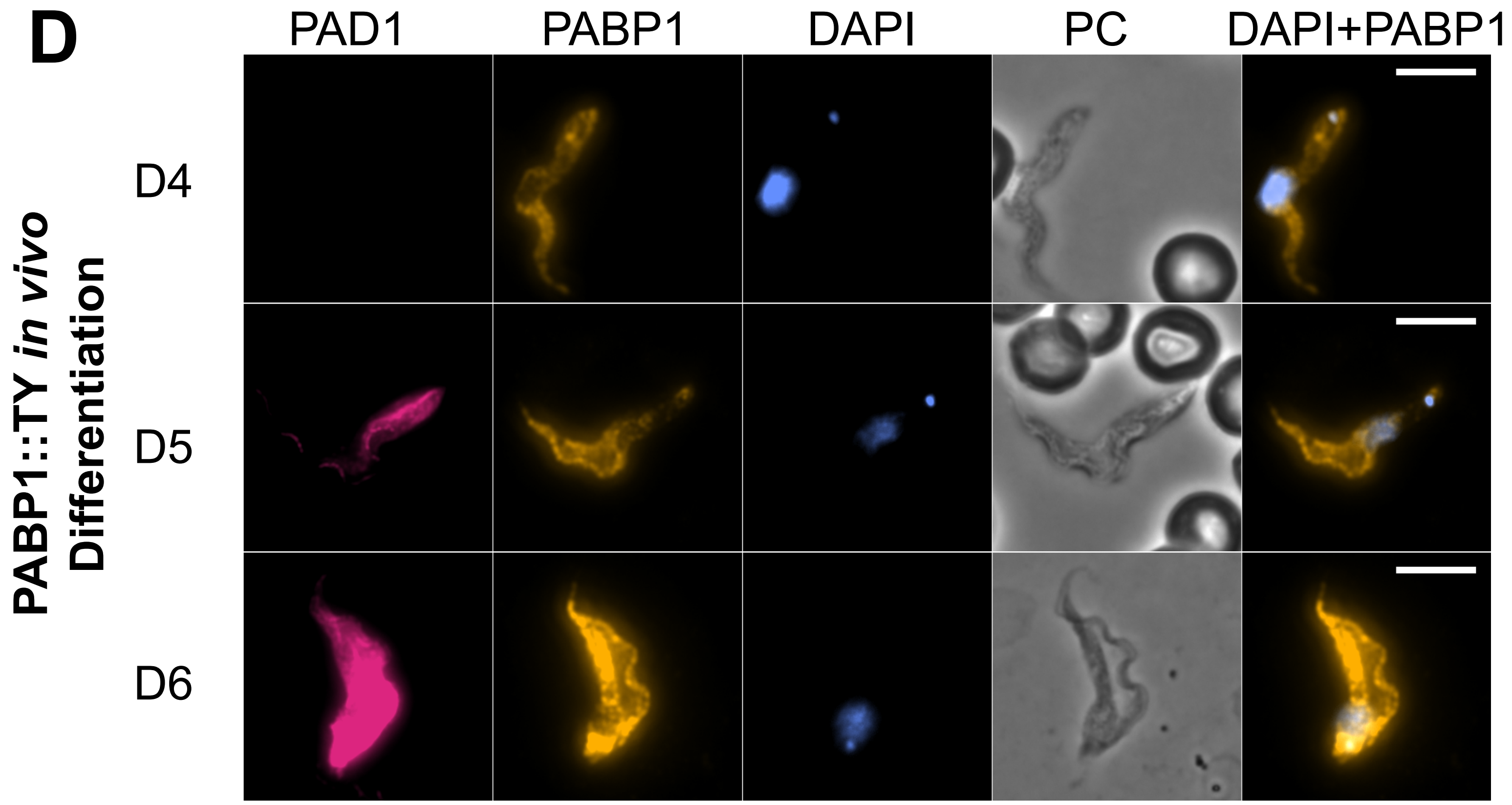

Supplementary figure 3 legend.

Starvation stress granules and differentiation granules are different. A) Immunofluorescence of bloodstream slender line PABP2::TYmNG exposed to glucose starvation for 60 min. Immunofluorescence of blood-smears of mice infected with B) Colocalization analysis of PABP2 and Alba 3/4 proteins during in vitro glucose starvation. The PABP2::TYmNG (AnTat1.1 J1339 PABP2::TYmNG) cell line was used and PABP2 localisation was revealed using the anti-Ty antibody (green in merge) and Alba 3/4 was revealed using the anti-alba 3 antibody also recognising Alba 4 (red in merge). Z-stack projection of a representative parasite is presented. Manders correlation was used to determine the percentage of pixels of Alba 3/4 colocalizing with Ty::PABP2 (left panel) and of Ty::PABP2 colocalizing with Alba 3/4 (right panel). Boxplots represent the interquartile range (IQR) from the 1st (25th percentile, Q1) to the 3rd (75th percentile, Q3) quartile, the median and whiskers indicate the maximum (Q3 + 1.5*IQR) and minimum (Q1 - 1.5*IQR) values. Individual data points are shown using overlaid dot plots. C) PABP2::TYmNG or D) PABP1::TYmNG tagged cell lines from D4 to D6 post-infection. PAD1 expression is revealed using anti-PAD1 antibody (PAD1, false coloured Pink), PABP1 or PABP2 localisation is revealed by anti-Ty antibody (PABP1/PABP2, false coloured Yellow), nucleus and kinetoplast using DAPI (false coloured Blue). PC Phase contrast. Acquisition settings were kept constant to detect weak positive cells, leading to saturation of the PAD1 signal on late time points. Scale bar = 10µm. Arrows highlight starvation granules.

Supplementary figure 4

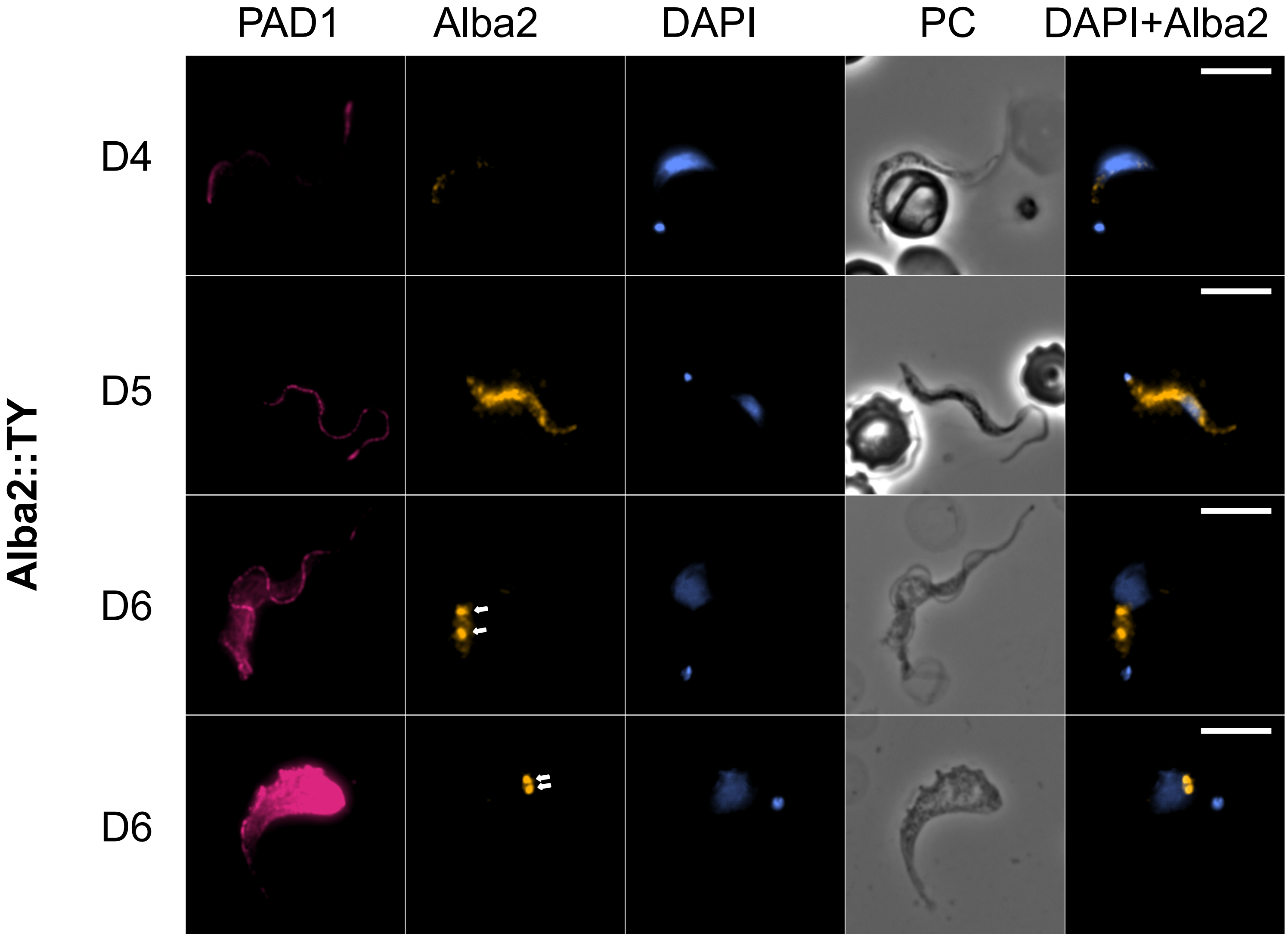

Supplementary figure 4 legend.

Alba 2 is targeted to differentiation granules at a late time point during differentiation. Immunofluorescence of blood-smears of mice infected with the Alba2::TYmNG tagged cell line from day 4 (D4) to day 6 (D6) post-infection. PAD1 expression is detected using anti-PAD1 antibody (PAD1, false coloured Pink), Alba 2 localisation is revealed by anti-Ty antibody (Alba2, false coloured Yellow), nucleus and kinetoplast using DAPI (false coloured Blue). PC Phase contrast. Acquisition settings were kept constant to detect weak positive cells, leading to saturation of the PAD1 signal on late time points. Scale bar = 10μm. Arrows highlight differentiation granules.

Supplementary figure 5

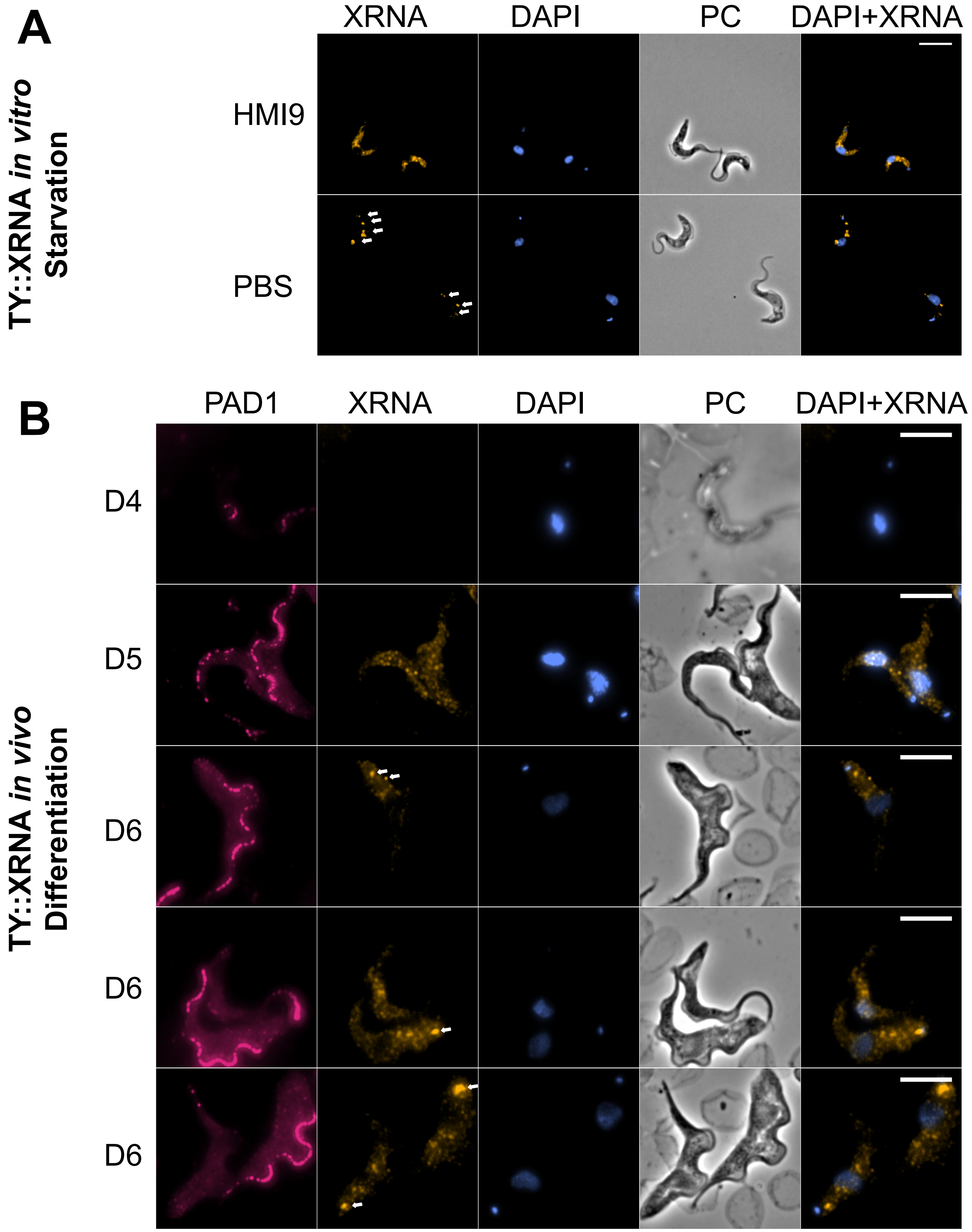

Supplementary figure 5

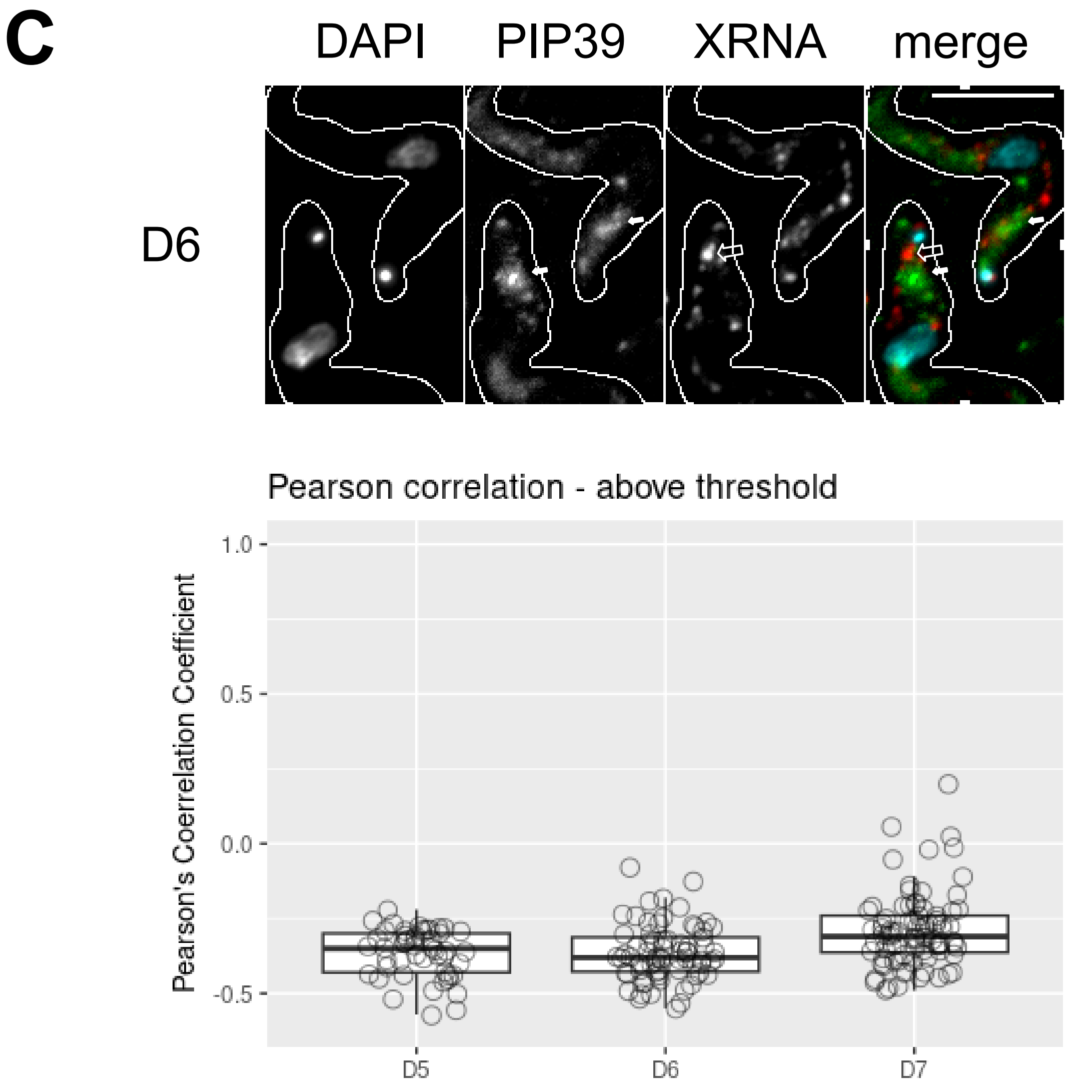

Supplementary figure 5 legend.

XRNA is targeted to differentiation granules during quorum-sensing driven differentiation in vivo and to stress granules during glucose starvation in vitro. A) Immunofluorescence of bloodstream slender line TYmNG::XRNA exposed to glucose starvation for 30 min in PBS. B) Immunofluorescence of blood-smears of mice infected with the TYmNG::XRNA tagged cell line from D4 to D6 post-infection. PAD1 expression is revealed using anti-PAD1 antibody (PAD1, false coloured Pink), XRNA localisation is revealed by anti-Ty antibody (XRNA, false coloured Yellow), nucleus and kinetoplast using DAPI (false coloured Blue). PC Phase contrast. Scale bar = 10µm. Arrows highlight starvation granules (panel A) differentiation granules (panel B). C) Immunofluorescence of blood-smears of mice infected with the TYmNG::XRNA tagged cell line to investigate the colocalization of the tagged XRNA with TbPIP39. The upper panel is a representative image of the localisation of TbPIP39 (antibody anti-TbPIP39, false coloured green) and XRNA (Antibody anti-Ty, false coloured red) at D6 post-infection. Nucleus and kinetoplast are revealed using DAPI (false coloured Blue). Plain arrows indicate the STuRN localisation of PIP39, and the empty arrow the localisation of the proximal XRNA+ve granule. Scale bar = 10µm. The lower panel present the pearson correlation coefficient of both proteins from D5 to D7 post infection. Boxplots represent the interquartile range (IQR) from the 1st (25th percentile, Q1) to the 3rd (75th percentile, Q3) quartile, the median and whiskers indicate the maximum (Q3 + 1.5*IQR) and minimum (Q1 - 1.5*IQR) values. Individual data points are shown using overlaid dot plots.

Supplementary figure 6

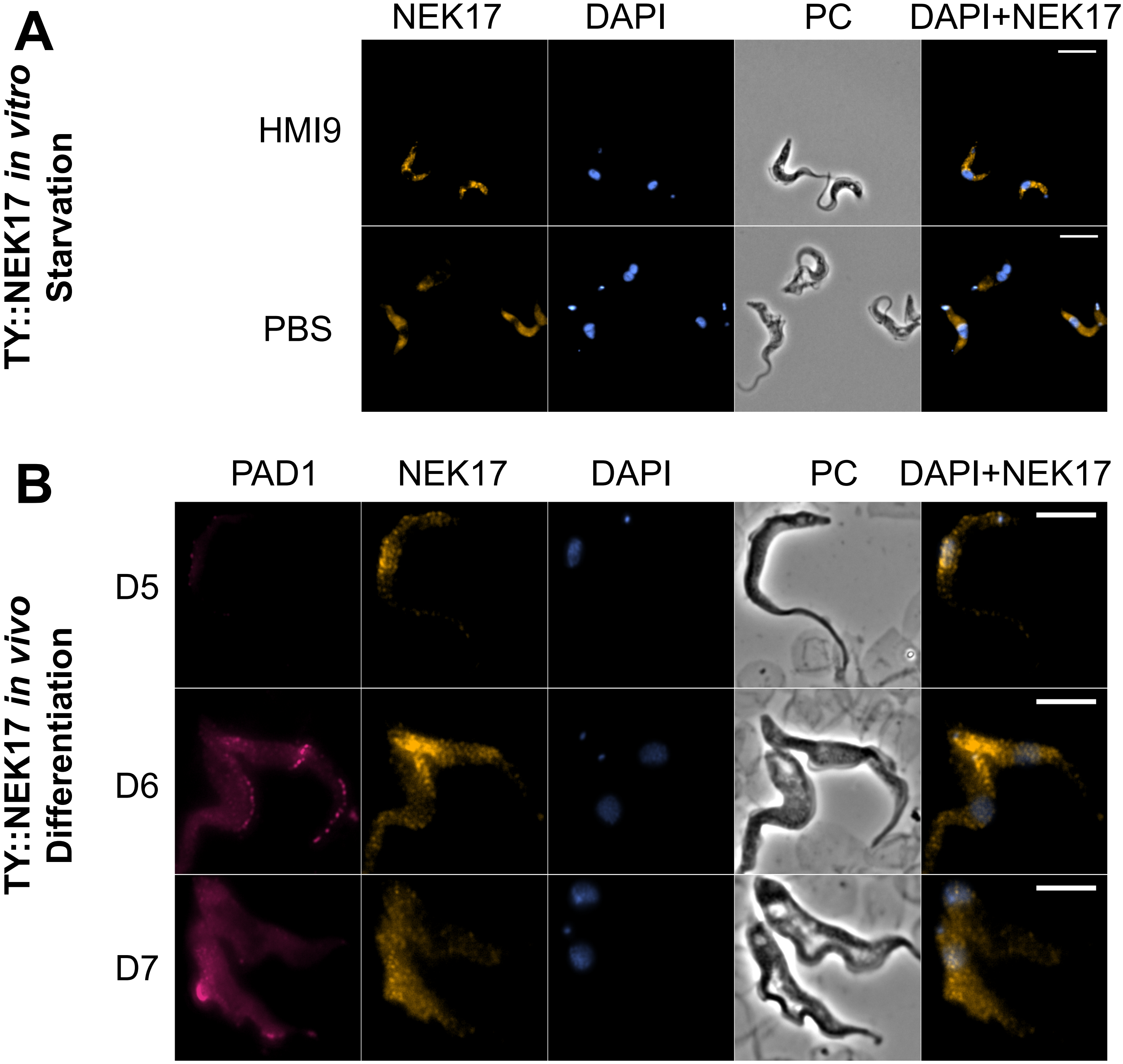

Supplementary figure 6 legend.

NEK17 remains cytoplasmic during quorum-sensing driven differentiation in vivo and glucose starvation in vitro. A) Immunofluorescence of bloodstream slender line TYmNG::NEK17 exposed to glucose starvation for 30 min in PBS. B) Immunofluorescence of blood-smears of mice infected with the TYmNG::NEK17 tagged cell line from D4 to D6 post-infection. PAD1 expression is revealed using anti-PAD1 antibody (PAD1, false coloured Pink), NEK17 localisation is revealed by anti-Ty antibody (NEK17, false coloured Yellow), nucleus and kinetoplast using DAPI (false coloured Blue). PC Phase contrast. Scale bar = 10μm.

Supplementary figure 7

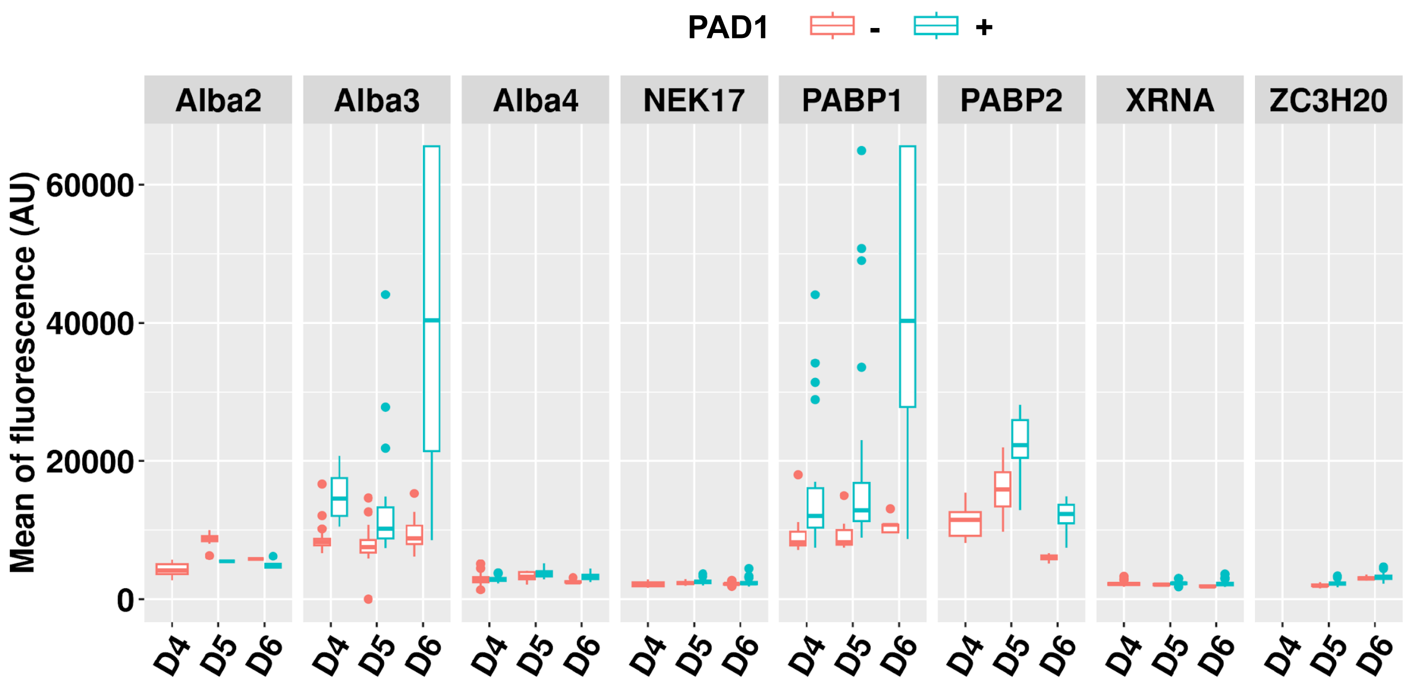

Supplementary figure 7 legend.

Measurement of mean fluorescence intensity as a proxy for protein abundance per parasite. The mean of fluorescence intensity was measured for each parasite expressing the different tagged proteins during the time course of the infection using the Ty1-tag signal. Measurement was performed in both PAD1 negative (-, red) and PAD1 positive (+, blue) cells. Boxplots represent the interquartile range (IQR) from the 1st (25th percentile, Q1) to the 3rd (75th percentile, Q3) quartile, the median and whiskers indicate the maximum (Q3 + 1.5*IQR) and minimum (Q1 - 1.5*IQR) values. Potential outliers are represented by points.

Supplementary figure 8

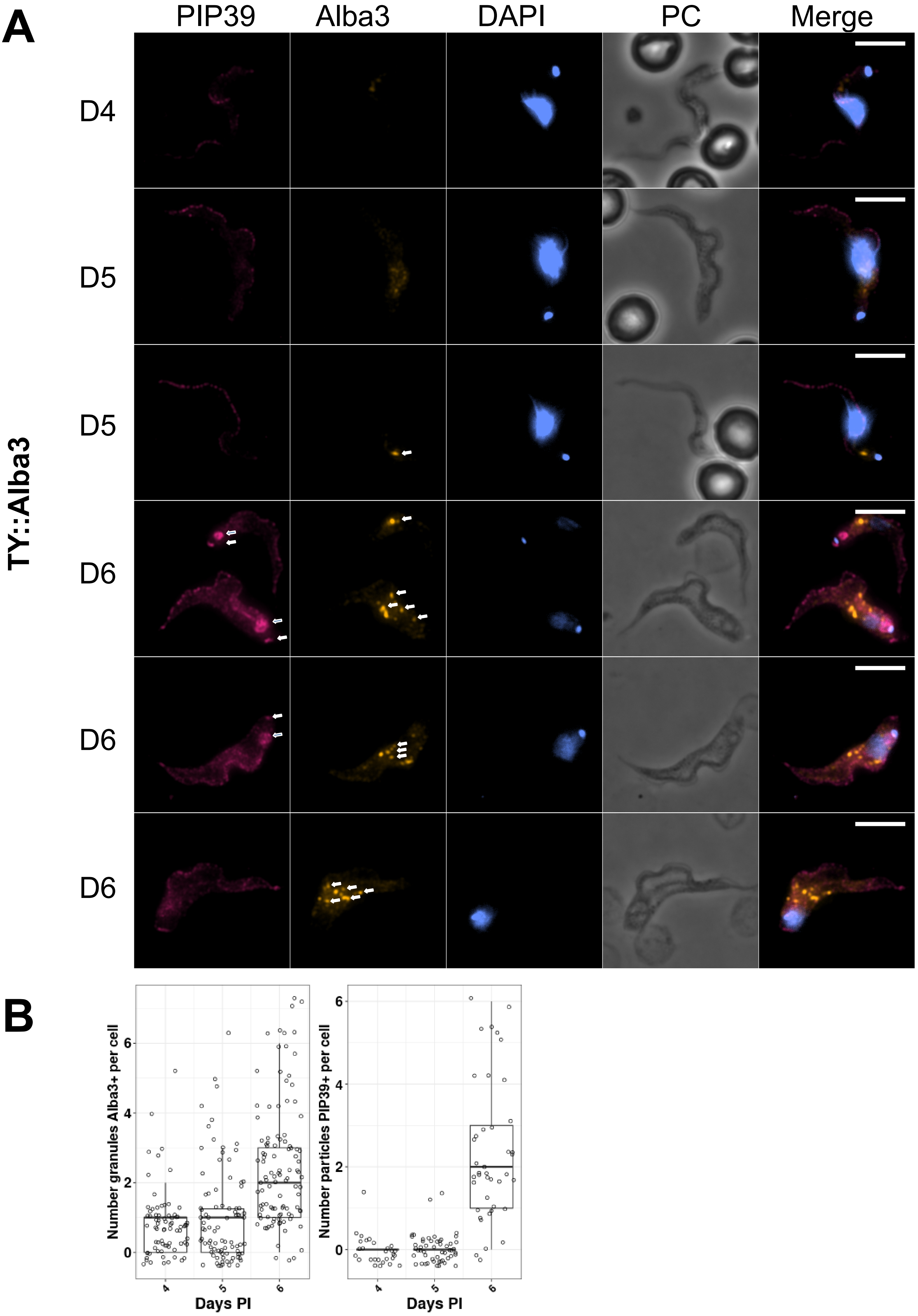

Supplementary figure 8 legend.

Alba 3+ve differentiation granules are not targeted to the STuRN. A) Immunofluorescence of blood-smears of mice infected with the TYmNG::Alba 3 tagged cell line from day 4 (D4) to day 6 (D6) post-infection. PIP39 localisation is detected using anti-PIP39 antibody (PIP39, false coloured Pink), Alba 3 localisation is revealed by anti-Ty antibody (Alba 3, false coloured Yellow), nucleus and kinetoplast using DAPI (false coloured Blue). PC Phase contrast. Scale bar = 10μm. Arrows highlight differentiation granules. B) Quantification of granular structures that are Ty tag positive (left panel) and PIP39 positive (right panel) during the time course of the infection. Boxplots represent the interquartile range (IQR) from the 1st (25th percentile, Q1) to the 3rd (75th percentile, Q3) quartile, the median and whiskers indicate the maximum (Q3 + 1.5*IQR) and minimum (Q1 - 1.5*IQR) values. Individual data points are shown using overlaid dot plots.

Supplementary figure 9

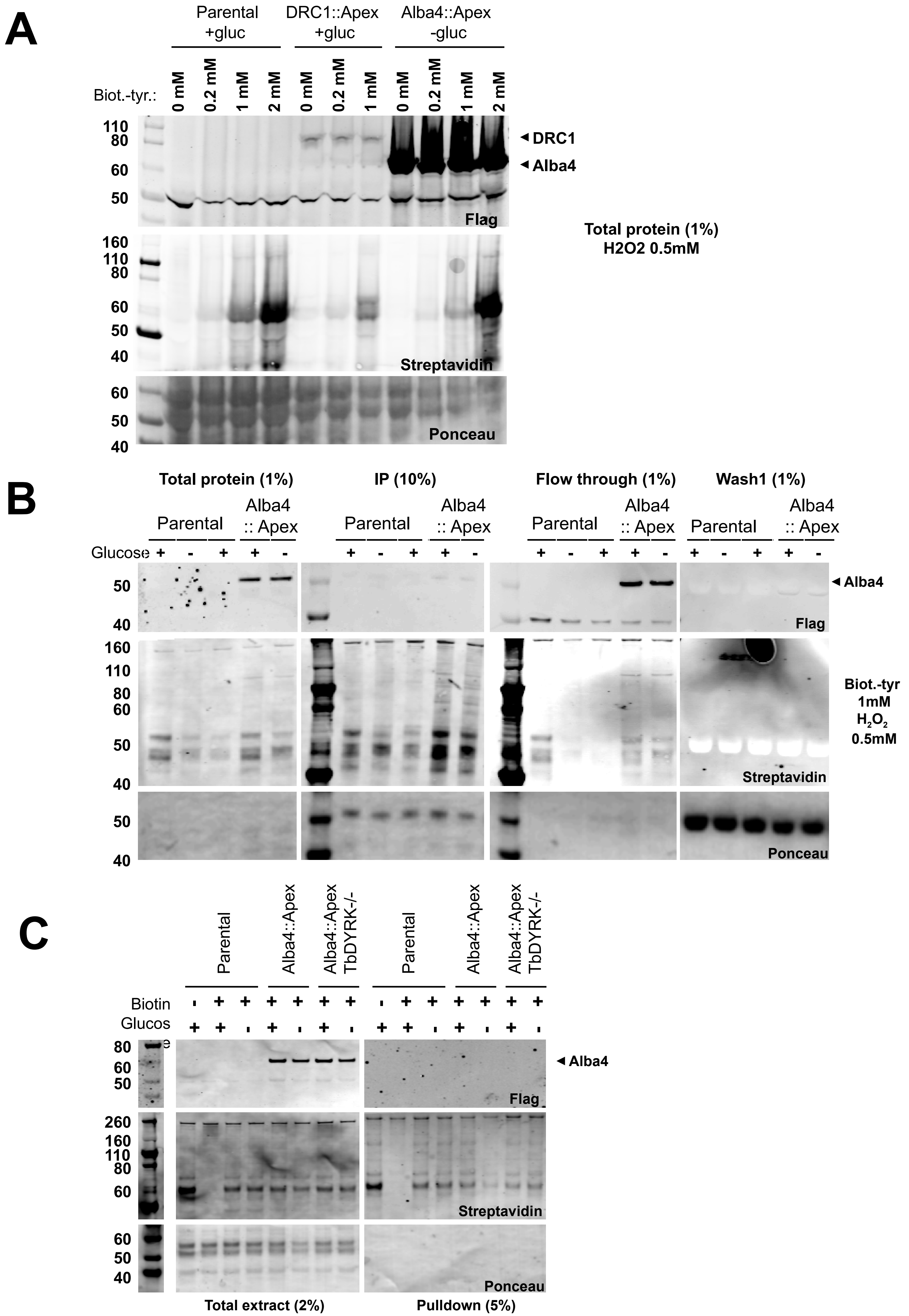

Supplementary figure 9 legend.

Western blot analysis for the validation of proximity labelling conditions using DRC1 and Alba 4 as bait proteins. Cell Line: Parental = AnTat1.1 J1339, DRC1::Apex = AnTat1.1 J1339 DRC1::Apex2-flag, Alba 4::Apex = AnTat1.1 J1339 Alba 4::Apex2-flag. Western blot revealed using anti-Flag (top panel), biotinylated protein using streptavidin, with loading controlled using ponceau staining of the membrane (lower panel). Black arrowheads indicate the expected sizes of DRC1 and Alba 4. Conditions tested are A) +/-glucose in presence of increased concentration of Biotin-tyramide (Biot.-tyr., from 0mM to 2mM), B-C) Biotin-tyramide 1mM, H2O2 0.5mM with pulldown (IP) of biotinylated proteins presented as well as flow-through and wash of beads indicated in respective panels. ‘Percent’ represents the percent of volume loaded of the total volume available.

Supplementary figure 10

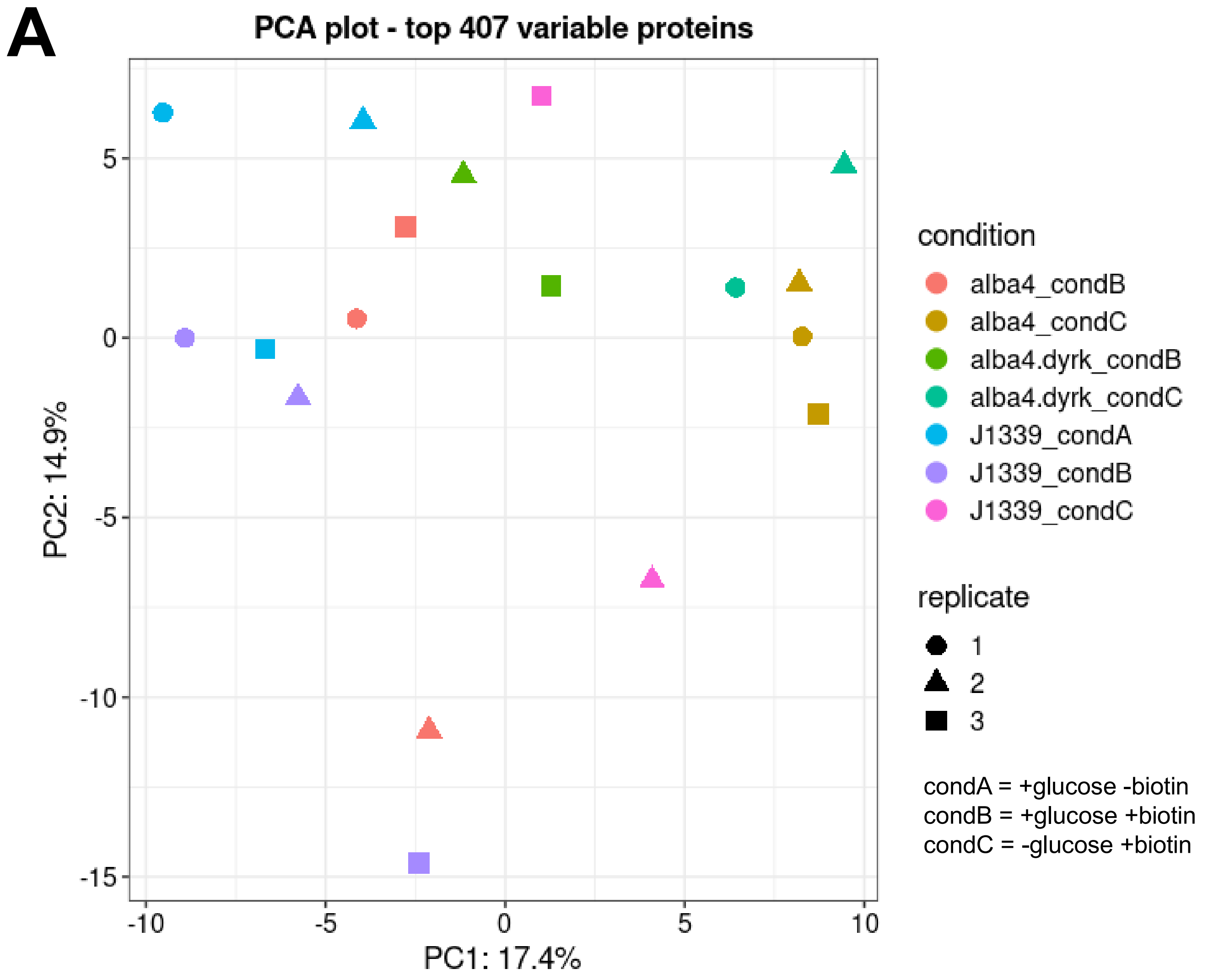

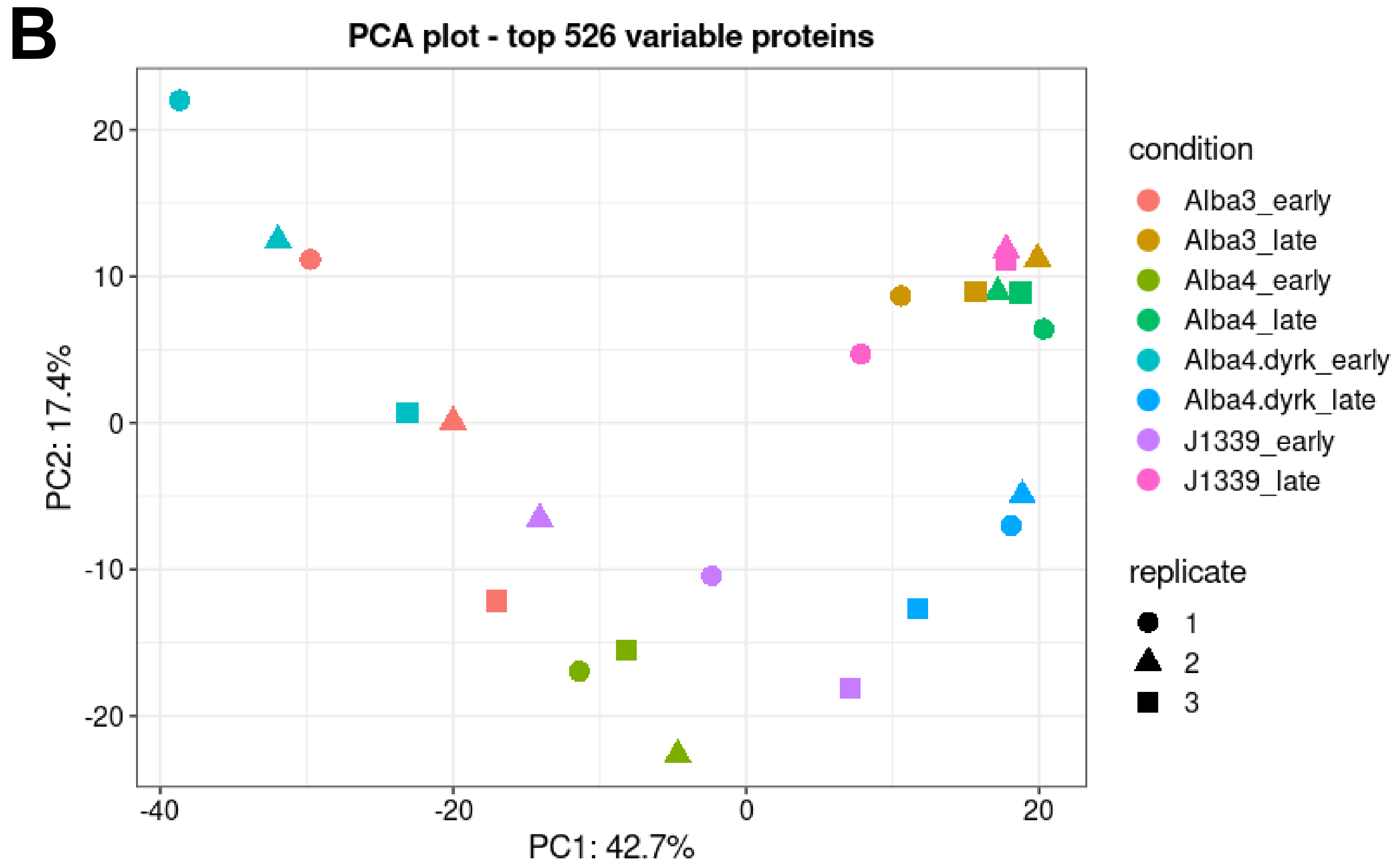

Supplementary figure 10 legend.

PCA plots of the proximity labelling samples used for quantification. A) Samples obtained from in vitro glucose starvation assay. condA = +glucose-biotin, condB = +glucose+biotin, condC = -glucose+biotin. B) samples obtained after parasite purification from infected mice at the early or late time point. Alba3 = AnTat1.1 J1339 Apex2-flag::Alba 3, Alba4 = AnTat1.1 J1339 Alba 4::Apex2-flag, Alba4.dyrk = AnTat1.1 TbDYRK-/- Alba 4::Apex2-flag. The percentages represent the principal components (PCs) explaining the variance of the data analysed. 2 PCs are indicated, PC1 on the x-axis and PC2 on the y-axis. Together they explain 32.3 and 60.1 % of the variance of the starvation (A) and differentiation (B) proximity labelling datasets, respectively.

Supplementary figure 11

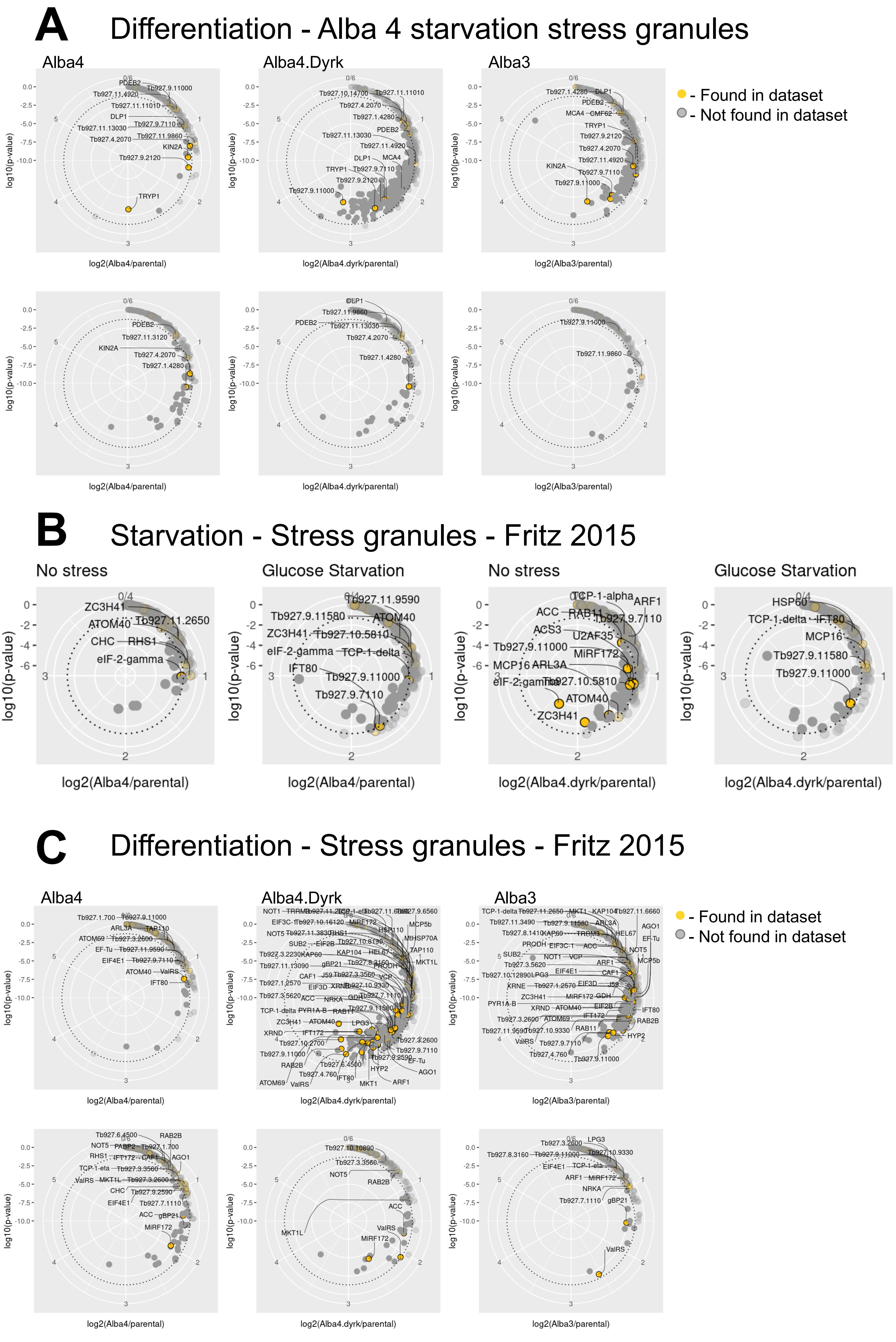

Supplementary figure 11 legend.

Dataset comparisons of granule composition under different conditions. A) Identification of differentiation granules protein common with starvation granules. B) Proteins identified in Alba 4+ve glucose starvation granules with common proteins identified in granules purified using the cytoskeleton as a sieve approach[1]. C) Proteins identified in differentiation granules Alba 3+ve and 4+ve that are common with proteins identified in granules purified using the cytoskeleton as a sieve approach[1].

Supplementary figure 12

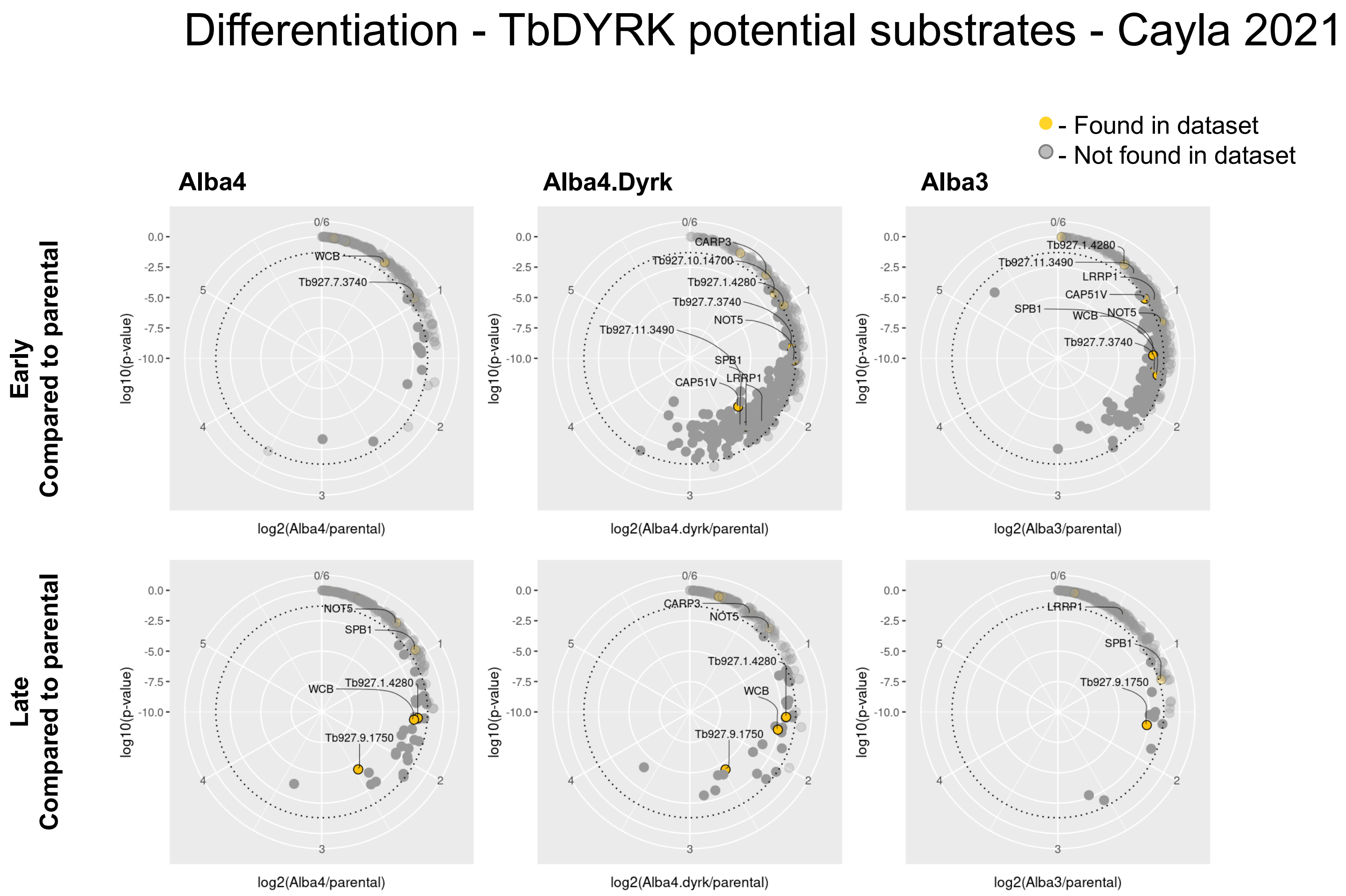

Supplementary figure 12 legend.

Dataset comparisons of granule composition with respect to potential TbDYRK substrates identified in Cayla et al., 2021[2]. Identification of differentiation granules proteins, potential substrates of TbDYRK.

Supplementary figure 13

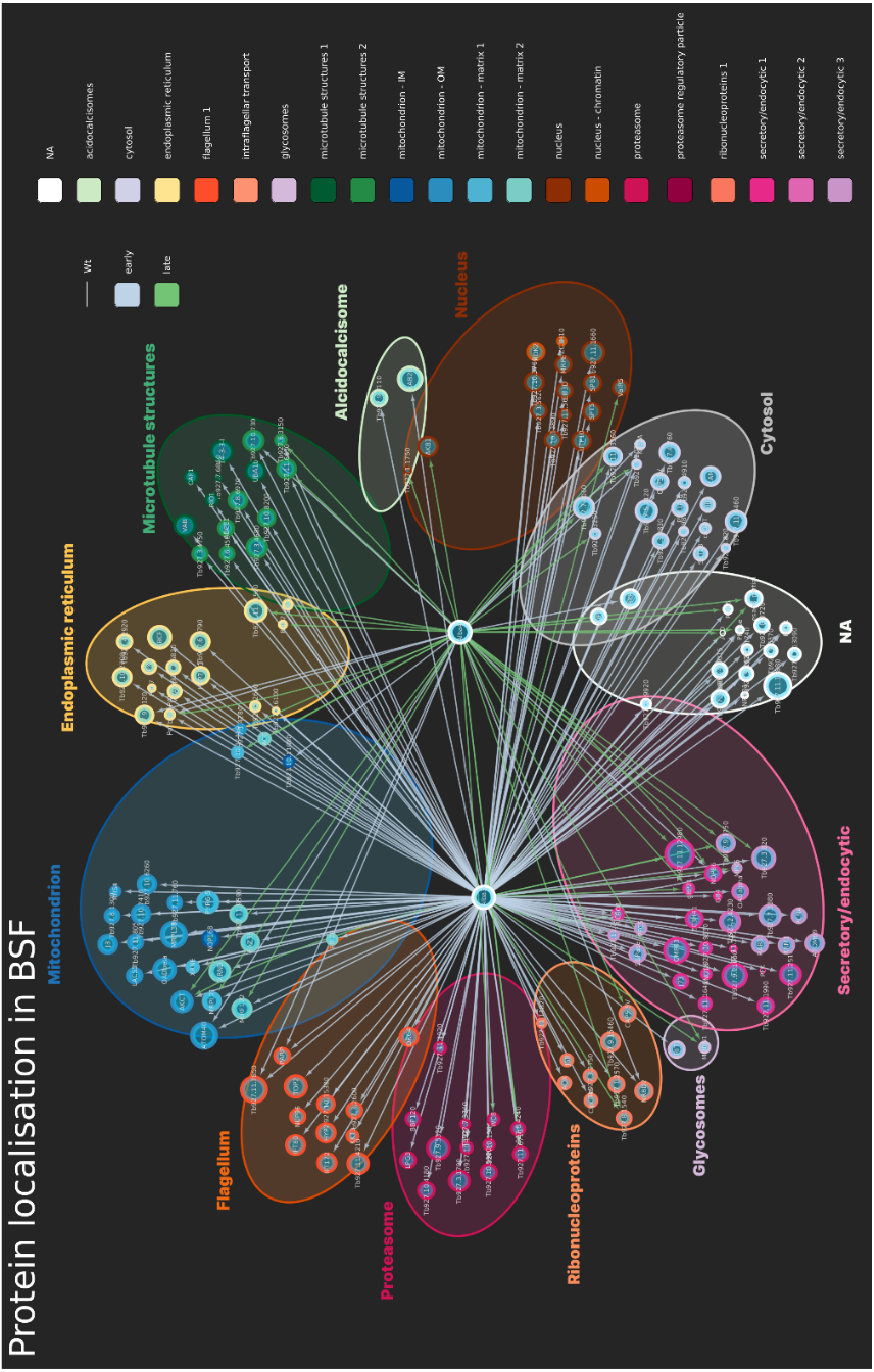

Supplementary figure 13 legend.

Network visualisation of the proteins proximal to Alba 3 and Alba 4 during the bloodstream differentiation. Early (light blue arrows) and Late (green arrows) time point are represented by the coloured arrows. Proteins were grouped and colour coded according to their localisation identified by Moloney et al[3]. The LOPIT dataset was generated on the bloodstream form of the parasite and therefore was used in our analysis instead of the Tryptag dataset that was generated at a different life cycle. Size of the circles represent the log2 fold change enrichment in the bait protein at a given time point compared to the parental cell line. For visualisation purposes, in the case a protein is identified more than once, only the biggest log2 fold change enrichment is represented.

References

1. Fritz, M. et al. Novel insights into RNP granules by employing the trypanosome's microtubule skeleton as a molecular sieve. Nucleic Acids Res. 43, 8013–8032 (2015).

2. Cayla, M., McDonald, L., MacGregor, P. & Matthews, K. An atypical DYRK kinase connects quorum-sensing with posttranscriptional gene regulation in Trypanosoma brucei. eLife 9, 1–33 (2020).

3. Moloney, N. M. et al. Mapping diversity in African trypanosomes using high resolution spatial proteomics. Nat. Commun. 14, 4401 (2023).
