# Supplementary material for: Differentiation granules, a dynamic regulator of T. brucei development
Source: Nat Commun. 2024 Apr 6;15:2972. doi: 10.1038/s41467-024-47309-1 (PMC10998879; doi:10.1038/s41467-024-47309-1)
Supplement: Supplementary file 3 — Description of Additional Supplementary Files [file 41467_2024_47309_MOESM3_ESM.pdf]

Description of Additional Supplementary Files

TITLE:

Supplementary Data 1

Description:

Provides the primers list used in this study, the star method table as well as the p-value calculation and numbers of cell counted for the results presented in Figure 2. The non-parametric Wilcoxon test was used to compare means of 2 independent samples. The null hypothesis states that the distributions of both populations are identical. Assuming that the responses are continuous, the alternative is restricted to a shift in location. Data used to produce figures 2, 4a, 4b and 5b are provided in the corresponding spreadsheets. Signal localisation name: DifCyt = Diffuse, GranPeriNu = Granules Perinuclear, PostNuGran = Granules Posterior, AntNuGran = Granules Anterior, STURN = proximal STURN/FP.

TITLE:

Supplementary Data 2

Description:

Results of the proximity proteomes obtained from glucose starvation and during the quorum sensing differentiation process. Statistical enrichment analysis was performed using two-sided moderated t-statistic as described in the Methods section. Data present the p-values and ratio obtained for the given comparison as well as information on the protein localisation (Tryptag, DeepLoc), the presence of low-complexity regions (lcr_005)[1], GO term analysis and their identification in published datasets (Substrate of TbDYRK = Cayla2020[2], Presence in stress granules = Fritz2015[3], Identified as components of the quorum sensing pathway = Sif, Proteins upregulated in Stumpy and Slender =

Dejeung2016upSS/SL[4], Proteins identified as presenting expression regulation in Alba3 knock-out in methyl cellulose-derived stumpy or procyclic = Bevka2023alba3SS/Proc[5], Genes for which mRNA has been identified as cell cycle regulated = CCR[6]). condB = +glucose, condC = -glucose, early = early time point during differentiation, late = late time point during differentiation.

REFERENCES

1. Cayla, M., Matthews, K. R. & Ivens, A. C. A global analysis of low-complexity regions in the Trypanosoma brucei proteome reveals enrichment in the C-terminus of nucleic acid binding proteins providing potential targets of phosphorylation. *Wellcome Open Res.* **5**, 219 (2020).
2. Cayla, M., McDonald, L., MacGregor, P. & Matthews, K. An atypical DYRK kinase connects quorum-sensing with posttranscriptional gene regulation in Trypanosoma brucei. *eLife* **9**, 1–33 (2020).
3. Fritz, M. *et al.* Novel insights into RNP granules by employing the trypanosome's microtubule skeleton as a molecular sieve. *Nucleic Acids Res.* **43**, 8013–8032 (2015).
4. Dejung, M. *et al.* Quantitative Proteomics Uncovers Novel Factors Involved in Developmental Differentiation of Trypanosoma brucei. *PLoS Pathog.* **12**, (2016).
5. Bevkal, S. *et al.* An Alba-domain protein required for proteome remodelling during trypanosome differentiation and host transition. *PLoS Pathog.* **17**, 1–30 (2021).
6. Briggs, E. M., Rojas, F., McCulloch, R., Matthews, K. R. & Otto, T. D. Single-cell transcriptomic analysis of bloodstream Trypanosoma brucei reconstructs cell cycle progression and developmental quorum sensing. *Nat. Commun.* **12**, 5268 (2021).
