# Supplementary material for: Differentiation granules, a dynamic regulator of T. brucei development
Source: Nat Commun. 2024 Apr 6;15:2972. doi: 10.1038/s41467-024-47309-1 (PMC10998879; doi:10.1038/s41467-024-47309-1)
Supplement: Supplementary file 6 — Reporting Summary [file 41467_2024_47309_MOESM6_ESM.pdf]

nature portfolio

Corresponding author(s): Mathieu Cayla

Last updated by author(s): 12/03/2024

Reporting Summary

Nature Portfolio wishes to improve the reproducibility of the work that we publish. This form provides structure for consistency and transparency in reporting. For further information on Nature Portfolio policies, see our Editorial Policies and the Editorial Policy Checklist.

Statistics

For all statistical analyses, confirm that the following items are present in the figure legend, table legend, main text, or Methods section.

n/a	Confirmed	
☐	☒	The exact sample size (*n*) for each experimental group/condition, given as a discrete number and unit of measurement
☐	☒	A statement on whether measurements were taken from distinct samples or whether the same sample was measured repeatedly
☐	☒	The statistical test(s) used AND whether they are one- or two-sided<br>*Only common tests should be described solely by name; describe more complex techniques in the Methods section.*
☒	☐	A description of all covariates tested
☐	☒	A description of any assumptions or corrections, such as tests of normality and adjustment for multiple comparisons
☐	☒	A full description of the statistical parameters including central tendency (e.g. means) or other basic estimates (e.g. regression coefficient) AND variation (e.g. standard deviation) or associated estimates of uncertainty (e.g. confidence intervals)
☐	☒	For null hypothesis testing, the test statistic (e.g. *F*, *t*, *r*) with confidence intervals, effect sizes, degrees of freedom and *P* value noted<br>*Give P values as exact values whenever suitable.*
☒	☐	For Bayesian analysis, information on the choice of priors and Markov chain Monte Carlo settings
☒	☐	For hierarchical and complex designs, identification of the appropriate level for tests and full reporting of outcomes
☒	☐	Estimates of effect sizes (e.g. Cohen's *d*, Pearson's *r*), indicating how they were calculated

Our web collection on statistics for biologists contains articles on many of the points above.

Software and code

Policy information about availability of computer code

Data collection	Immunofluorescence: Zeiss Axio Imager Z2 mounted with a Prime BSI (Teledyne Photometrics) camera.<br>Mass spectrometry: Orbitrap Fusion™ Lumos™ Tribrid™ Mass Spectrometer (Thermo Fisher Scientific, UK) both coupled on-line, to an Ultimate 3000 HPLC (Dionex, Thermo Fisher Scientific, UK)
Data analysis	µManager 2.0, Fiji (ImageJ 1.54f),MaxQuant (1.6.1.0), R (3.6.3), R studio (2022.07.2 Build 576), DEP (1.8.0), Blast, Eukaryotic Linear Motif resource (2022 release), Jalview (2.11.2.0) LeishGEdit, MUSCLE, OrthoMCL (release 6.18), Pfam, TriTrypDB (release 65)

For manuscripts utilizing custom algorithms or software that are central to the research but not yet described in published literature, software must be made available to editors and reviewers. We strongly encourage code deposition in a community repository (e.g. GitHub). See the Nature Portfolio guidelines for submitting code & software for further information.

Data

Policy information about availability of data

All manuscripts must include a data availability statement. This statement should provide the following information, where applicable:

- Accession codes, unique identifiers, or web links for publicly available datasets
- A description of any restrictions on data availability
- For clinical datasets or third party data, please ensure that the statement adheres to our policy

The raw proteomic data generated in this study have been deposited in the Pride database from the ProteomeXchange consortium with identifier PXD045841 [add

hyp]. The processed proteomic data generated in this study are provided in the Supplementary Data file 2.
The data generated from images analysis used in the figures 2, 4, 5b have been deposited in the XXX database under the accession code XXX [link].

Research involving human participants, their data, or biological material

Policy information about studies with human participants or human data. See also policy information about sex, gender (identity/presentation), and sexual orientation and race, ethnicity and racism.

Reporting on sex and gender	This study did not involve human participants, their data or biological material.
Reporting on race, ethnicity, or other socially relevant groupings	This study did not involve human participants, their data or biological material.
Population characteristics	This study did not involve human participants, their data or biological material.
Recruitment	This study did not involve human participants, their data or biological material.
Ethics oversight	This study did not involve human participants, their data or biological material.

Note that full information on the approval of the study protocol must also be provided in the manuscript.

Field-specific reporting

Please select the one below that is the best fit for your research. If you are not sure, read the appropriate sections before making your selection.

[x] Life sciences [ ] Behavioural & social sciences [ ] Ecological, evolutionary & environmental sciences

For a reference copy of the document with all sections, see nature.com/documents/nr-reporting-summary-flat.pdf

Life sciences study design

All studies must disclose on these points even when the disclosure is negative.

Sample size	n=3 unless indicated otherwise. Variance across experiments was low so explicit power calculation was not considered necessary. For the analysis of phenotypes resulting from mice infection of genetically modified parasites, n=3 animals are considered sufficient to obtain results suitable to provide a power of 80% to detect 10% differences.
Data exclusions	For the proteomic experiments, Peptide and protein identifications were filtered to 1% FDR. Samples not used for further analysis, due to poor amount of proteins detected, are indicated in the text.
Replication	Experiments were replicated at least 3 times on different days. All experiment replication were successful and used for the study.
Randomization	Random allocation with regard to covariates is not applicable to the experiments carried out. This is because MF1 mice are highly homogenous in sex (females), size (range = 20-30g, average = 25g) and age (between 6-10 weeks, average 8 weeks) in our design of experiment. Furthermore, infection outcome are highly reproducible, with In a classical infection the three outcomes of interest measured in these experiments, i.e. parasitaemia, differentiation and cell cycle stage, present a variation inferior to 5% between samples of a same group.
Blinding	Blinding was not relevant for the experiments carried out in this study. Investigators are aware of the group allocation (not blinded) as group of mice are homogenous in sex (females), size (range = 20-30g, average = 25g) and age (between 6-10 weeks, average 8 weeks).

Behavioural & social sciences study design

All studies must disclose on these points even when the disclosure is negative.

Study description	*Briefly describe the study type including whether data are quantitative, qualitative, or mixed-methods (e.g. qualitative cross-sectional, quantitative experimental, mixed-methods case study).*
Research sample	*State the research sample (e.g. Harvard university undergraduates, villagers in rural India) and provide relevant demographic information (e.g. age, sex) and indicate whether the sample is representative. Provide a rationale for the study sample chosen. For studies involving existing datasets, please describe the dataset and source.*
Sampling strategy	*Describe the sampling procedure (e.g. random, snowball, stratified, convenience). Describe the statistical methods that were used to predetermine sample size OR if no sample-size calculation was performed, describe how sample sizes were chosen and provide a rationale for why these sample sizes are sufficient. For qualitative data, please indicate whether data saturation was considered, and what criteria were used to decide that no further sampling was needed.*

Data collection	*Provide details about the data collection procedure, including the instruments or devices used to record the data (e.g. pen and paper, computer, eye tracker, video or audio equipment) whether anyone was present besides the participant(s) and the researcher, and whether the researcher was blind to experimental condition and/or the study hypothesis during data collection.*
Timing	*Indicate the start and stop dates of data collection. If there is a gap between collection periods, state the dates for each sample cohort.*
Data exclusions	*If no data were excluded from the analyses, state so OR if data were excluded, provide the exact number of exclusions and the rationale behind them, indicating whether exclusion criteria were pre-established.*
Non-participation	*State how many participants dropped out/declined participation and the reason(s) given OR provide response rate OR state that no participants dropped out/declined participation.*
Randomization	*If participants were not allocated into experimental groups, state so OR describe how participants were allocated to groups, and if allocation was not random, describe how covariates were controlled.*

Ecological, evolutionary & environmental sciences study design

All studies must disclose on these points even when the disclosure is negative.

Study description	*Briefly describe the study. For quantitative data include treatment factors and interactions, design structure (e.g. factorial, nested, hierarchical), nature and number of experimental units and replicates.*
Research sample	*Describe the research sample (e.g. a group of tagged Passer domesticus, all Stenocereus thurberi within Organ Pipe Cactus National Monument), and provide a rationale for the sample choice. When relevant, describe the organism taxa, source, sex, age range and any manipulations. State what population the sample is meant to represent when applicable. For studies involving existing datasets, describe the data and its source.*
Sampling strategy	*Note the sampling procedure. Describe the statistical methods that were used to predetermine sample size OR if no sample-size calculation was performed, describe how sample sizes were chosen and provide a rationale for why these sample sizes are sufficient.*
Data collection	*Describe the data collection procedure, including who recorded the data and how.*
Timing and spatial scale	*Indicate the start and stop dates of data collection, noting the frequency and periodicity of sampling and providing a rationale for these choices. If there is a gap between collection periods, state the dates for each sample cohort. Specify the spatial scale from which the data are taken*
Data exclusions	*If no data were excluded from the analyses, state so OR if data were excluded, describe the exclusions and the rationale behind them, indicating whether exclusion criteria were pre-established.*
Reproducibility	*Describe the measures taken to verify the reproducibility of experimental findings. For each experiment, note whether any attempts to repeat the experiment failed OR state that all attempts to repeat the experiment were successful.*
Randomization	*Describe how samples/organisms/participants were allocated into groups. If allocation was not random, describe how covariates were controlled. If this is not relevant to your study, explain why.*
Blinding	*Describe the extent of blinding used during data acquisition and analysis. If blinding was not possible, describe why OR explain why blinding was not relevant to your study.*

Did the study involve field work? ☐ Yes ☒ No

Field work, collection and transport

Field conditions	This study did not involve field work.
Location	This study did not involve field work.
Access & import/export	This study did not involve field work.
Disturbance	This study did not involve field work.

Reporting for specific materials, systems and methods

We require information from authors about some types of materials, experimental systems and methods used in many studies. Here, indicate whether each material, system or method listed is relevant to your study. If you are not sure if a list item applies to your research, read the appropriate section before selecting a response.

Materials & experimental systems

n/a	Involved in the study
	☒ Antibodies
	☒ Eukaryotic cell lines
☒	Palaeontology and archaeology
	☒ Animals and other organisms
☒	Clinical data
☒	Dual use research of concern
☒	Plants

Methods

n/a	Involved in the study
☒	ChIP-seq
☒	Flow cytometry
☒	MRI-based neuroimaging

Antibodies

Antibodies used

Anti-Ty1 epitope tag specific BB2 antibody - Bastin et al., 1996; hybridoma cell line a gift of Keith Gull, Oxford University/available through Thermofisher - Cat#MA5-23513; RRID:AB_2610644 - Hybrydome mouse monoclonal, clone BB2, WB (1:20), IF (1:5)
Anti-Mouse AlexaFluor 488 - molecular probes - Cat# A1101 - Goat polyclonal, IF (1:500)
Anti-Mouse AlexaFluor 568 - abcam - Cat# ab175701 - Goat polyclonal, IF (1:500)
Anti-Rabbit AlexaFluor 488 - invitrogen - Cat# A1108 - Goat polyclonal, IF (1:500)
Anti-Rabbit AlexaFluor 568 - invitrogen - Cat# A11036 - Goat polyclonal, IF (1:500)
IRDye 680 anti-mouse secondary antibody - Li-Cor - Cat#P/N 925-68070; RRID:AB_2651128 - Goat polyclonal, IF (1:5000)
IRDye 800CW anti-Mouse IgG (H + L) secondary antibody - Li-Cor - Cat#P/N 925-32210; RRID:AB_2687825 - Goat polyclonal, IF (1:5000)
Anti-PAD1 - Dean et al., 2009 - N/A - Rabbit polyclonal, WB (1:1000), IF (1:1000)
Anti-Flag (M2 monoclonal) - sigma aldrich - Cat# F1804 - Mouse monoclonal, WB (1:2000), IF (1:200)

Validation

Validation of Anti-Ty1 epitope tag specific BB2 antibody was performed by Bastin et al., 1996
Validation of Anti-PAD1 was performed by Dean et al., 2009.
The AlexaFluor antibodies used in this study were validated as described : To minimize cross-reactivity, these goat anti-mouse IgG whole antibodies have been cross-adsorbed against human IgG and human serum. Cross-adsorption or pre-adsorption is a purification step to increase specificity of the antibody resulting in higher sensitivity and less background staining. The secondary antibody solution is passed through a column matrix containing immobilized serum proteins from potentially cross-reactive species. Only the nonspecific-binding secondary antibodies are captured in the column, and the highly specific secondaries flow through. The benefits of this extra step are apparent in multiplexing/multicolor-staining experiments (e.g., flow cytometry) where there is potential cross-reactivity with other primary antibodies or in tissue/cell fluorescent staining experiments where there are may be the presence of endogenous immunoglobulins.
The IRDye antibodies were validated as followed: tested by dot blot and and/or solid-phase adsorbed for minimal cross-reactivity with human, rabbit, goat, rat, and horse serum proteins, but may cross-react with immunoglobulins from other species. The conjugate has been specifically tested and qualified for Western blot and In-Cell Western™ Assay applications.
The anti-Flag antibody was optimized for single banded detection of FLAG fusion proteins in mammalian, plant, and bacterial expression systems.

Eukaryotic cell lines

Policy information about cell lines and Sex and Gender in Research

Cell line source(s)

Trypanosoma brucei EATRO 1125 AnTat1.1 J1339 (PMID: 30503212)
Trypanosoma brucei EATRO 1125 AnTat1.1 J1339_TbDYRK-/- (This study)
Trypanosoma brucei EATRO 1125 AnTat1.1 J1339_TbDYRK-/-_addback-NM (This study)
Trypanosoma brucei EATRO 1125 AnTat1.1 J1339_TbDYRK-/-_addback-S856G (This study)
Trypanosoma brucei EATRO 1125 AnTat1.1 J1339_ALBA4-/- (This study)
Trypanosoma brucei EATRO 1125 AnTat1.1 J1339_ALBA4::TymNG (This study)
Trypanosoma brucei EATRO 1125 AnTat1.1 J1339_ALBA4::Apex2-flag (This study)
Trypanosoma brucei EATRO 1125 AnTat1.1 J1339_ALBA3-/+ (This study)
Trypanosoma brucei EATRO 1125 AnTat1.1 J1339_TymNG::ALBA3 (This study)
Trypanosoma brucei EATRO 1125 AnTat1.1 J1339_flag-Apex2::ALBA3 (This study)
Trypanosoma brucei EATRO 1125 AnTat1.1 J1339_TbDYRK-/+_addback-S856G (This study)
Trypanosoma brucei EATRO 1125 AnTat1.1 J1339_TbDYRK-/+_addback-S856G (This study)
Trypanosoma brucei EATRO 1125 AnTat1.1 J1339_TymNG::XRNA (This study)
Trypanosoma brucei EATRO 1125 AnTat1.1 J1339_ZC3H20::TymNG (This study)
Trypanosoma brucei EATRO 1125 AnTat1.1 J1339_PABP1::TymNG (This study)
Trypanosoma brucei EATRO 1125 AnTat1.1 J1339_TymNG::PABP2 (This study)
Trypanosoma brucei EATRO 1125 AnTat1.1 J1339_PABP2::TymNG (This study)
Trypanosoma brucei EATRO 1125 AnTat1.1 J1339_RAB7::TymNG (This study)
Trypanosoma brucei EATRO 1125 AnTat1.1 J1339_TymNG::NEK17 (This study)
Trypanosoma brucei EATRO 1125 AnTat1.1 J1339_DRC1::Apex2-flag (This study)
Trypanosoma brucei EATRO 1125 AnTat1.1 J1339_ALBA2::TymNG (This study)
Trypanosoma brucei EATRO 1125 AnTat1.1 J1339_TbDYRK-/-_Alba4::TymNG (This study)

	Trypanosoma brucei EATRO 1125 AnTat1.1 J1339_TbDYRK-/-_Alba4::Apex2-flag (This study)
Authentication	Cell lines were cultured in the antibiotics each is stated to be resistant to in order to authenticate prior to beginning experiments. PCR with specific primers to TbDYRK, Alba3 and Alba4 genome regions were used to verify the corresponding KO cell lines. Endogenous tagging were verified by western blotting and IFA
Mycoplasma contamination	No mycoplasma testing was performed
Commonly misidentified lines (See ICLAC register)	No commonly misidentified cell lines were used in this study

Palaeontology and Archaeology

Specimen provenance	*Provide provenance information for specimens and describe permits that were obtained for the work (including the name of the issuing authority, the date of issue, and any identifying information). Permits should encompass collection and, where applicable, export.*
Specimen deposition	*Indicate where the specimens have been deposited to permit free access by other researchers.*
Dating methods	*If new dates are provided, describe how they were obtained (e.g. collection, storage, sample pretreatment and measurement), where they were obtained (i.e. lab name), the calibration program and the protocol for quality assurance OR state that no new dates are provided.*

☐ Tick this box to confirm that the raw and calibrated dates are available in the paper or in Supplementary Information.

Ethics oversight	*Identify the organization(s) that approved or provided guidance on the study protocol, OR state that no ethical approval or guidance was required and explain why not.*

Note that full information on the approval of the study protocol must also be provided in the manuscript.

Animals and other research organisms

Policy information about studies involving animals; ARRIVE guidelines recommended for reporting animal research, and Sex and Gender in Research

Laboratory animals	Mouse MF1, female, 8 weeks old
Wild animals	The study did not involve wild animals
Reporting on sex	Sex was not considered in the study design. Differentiation from the slender to stumpy form during the 1st peak of infection has not been reported to be affected by sex.
Field-collected samples	The study did not involve samples collected from the field
Ethics oversight	Animal experiments in this work were carried out in accordance with the local ethical approval requirements of the University of Edinburgh and the UK Home Office Animal (Scientific Procedures) Act (1986) under licence number PP2251183.

Note that full information on the approval of the study protocol must also be provided in the manuscript.

Clinical data

Policy information about clinical studies

All manuscripts should comply with the ICMJEguidelines for publication of clinical research and a completedCONSORT checklist must be included with all submissions.

Clinical trial registration	*Provide the trial registration number from ClinicalTrials.gov or an equivalent agency.*
Study protocol	*Note where the full trial protocol can be accessed OR if not available, explain why.*
Data collection	*Describe the settings and locales of data collection, noting the time periods of recruitment and data collection.*
Outcomes	*Describe how you pre-defined primary and secondary outcome measures and how you assessed these measures.*

Dual use research of concern

Policy information about dual use research of concern

Hazards

Could the accidental, deliberate or reckless misuse of agents or technologies generated in the work, or the application of information presented in the manuscript, pose a threat to:

No	Yes	
☒	☐	Public health
☒	☐	National security
☒	☐	Crops and/or livestock
☒	☐	Ecosystems
☒	☐	Any other significant area

Experiments of concern

Does the work involve any of these experiments of concern:

No	Yes	
☒	☐	Demonstrate how to render a vaccine ineffective
☒	☐	Confer resistance to therapeutically useful antibiotics or antiviral agents
☒	☐	Enhance the virulence of a pathogen or render a nonpathogen virulent
☒	☐	Increase transmissibility of a pathogen
☒	☐	Alter the host range of a pathogen
☒	☐	Enable evasion of diagnostic/detection modalities
☒	☐	Enable the weaponization of a biological agent or toxin
☒	☐	Any other potentially harmful combination of experiments and agents

Plants

Seed stocks	*Report on the source of all seed stocks or other plant material used. If applicable, state the seed stock centre and catalogue number. If plant specimens were collected from the field, describe the collection location, date and sampling procedures.*
Novel plant genotypes	*Describe the methods by which all novel plant genotypes were produced. This includes those generated by transgenic approaches, gene editing, chemical/radiation-based mutagenesis and hybridization. For transgenic lines, describe the transformation method, the number of independent lines analyzed and the generation upon which experiments were performed. For gene-edited lines, describe the editor used, the endogenous sequence targeted for editing, the targeting guide RNA sequence (if applicable) and how the editor was applied.*
Authentication	*Describe any authentication procedures for each seed stock used or novel genotype generated. Describe any experiments used to assess the effect of a mutation and, where applicable, how potential secondary effects (e.g. second site T-DNA insertions, mosiacism, off-target gene editing) were examined.*

ChIP-seq

Data deposition

☐ Confirm that both raw and final processed data have been deposited in a public database such as GEO.

☐ Confirm that you have deposited or provided access to graph files (e.g. BED files) for the called peaks.

Data access links<br>*May remain private before publication.*	*For "Initial submission" or "Revised version" documents, provide reviewer access links. For your "Final submission" document, provide a link to the deposited data.*
Files in database submission	*Provide a list of all files available in the database submission.*
Genome browser session<br>(e.g. UCSC)	*Provide a link to an anonymized genome browser session for "Initial submission" and "Revised version" documents only, to enable peer review. Write "no longer applicable" for "Final submission" documents.*

Methodology

Replicates	*Describe the experimental replicates, specifying number, type and replicate agreement.*
Sequencing depth	*Describe the sequencing depth for each experiment, providing the total number of reads, uniquely mapped reads, length of reads and whether they were paired- or single-end.*
Antibodies	*Describe the antibodies used for the ChIP-seq experiments; as applicable, provide supplier name, catalog number, clone name, and lot number.*

Peak calling parameters	*Specify the command line program and parameters used for read mapping and peak calling, including the ChIP, control and index files used.*
Data quality	*Describe the methods used to ensure data quality in full detail, including how many peaks are at FDR 5% and above 5-fold enrichment.*
Software	*Describe the software used to collect and analyze the ChIP-seq data. For custom code that has been deposited into a community repository, provide accession details.*

Flow Cytometry

Plots

Confirm that:

☐ The axis labels state the marker and fluorochrome used (e.g. CD4-FITC).

☐ The axis scales are clearly visible. Include numbers along axes only for bottom left plot of group (a 'group' is an analysis of identical markers).

☐ All plots are contour plots with outliers or pseudocolor plots.

☐ A numerical value for number of cells or percentage (with statistics) is provided.

Methodology

Sample preparation	*Describe the sample preparation, detailing the biological source of the cells and any tissue processing steps used.*
Instrument	*Identify the instrument used for data collection, specifying make and model number.*
Software	*Describe the software used to collect and analyze the flow cytometry data. For custom code that has been deposited into a community repository, provide accession details.*
Cell population abundance	*Describe the abundance of the relevant cell populations within post-sort fractions, providing details on the purity of the samples and how it was determined.*
Gating strategy	*Describe the gating strategy used for all relevant experiments, specifying the preliminary FSC/SSC gates of the starting cell population, indicating where boundaries between "positive" and "negative" staining cell populations are defined.*

☐ Tick this box to confirm that a figure exemplifying the gating strategy is provided in the Supplementary Information.

Magnetic resonance imaging

Experimental design

Design type	*Indicate task or resting state; event-related or block design.*
Design specifications	*Specify the number of blocks, trials or experimental units per session and/or subject, and specify the length of each trial or block (if trials are blocked) and interval between trials.*
Behavioral performance measures	*State number and/or type of variables recorded (e.g. correct button press, response time) and what statistics were used to establish that the subjects were performing the task as expected (e.g. mean, range, and/or standard deviation across subjects).*

Acquisition

Imaging type(s)	*Specify: functional, structural, diffusion, perfusion.*
Field strength	*Specify in Tesla*
Sequence & imaging parameters	*Specify the pulse sequence type (gradient echo, spin echo, etc.), imaging type (EPI, spiral, etc.), field of view, matrix size, slice thickness, orientation and TE/TR/flip angle.*
Area of acquisition	*State whether a whole brain scan was used OR define the area of acquisition, describing how the region was determined.*

Diffusion MRI ☐ Used ☐ Not used

Preprocessing

Preprocessing software	*Provide detail on software version and revision number and on specific parameters (model/functions, brain extraction, segmentation, smoothing kernel size, etc.).*

Normalization	*If data were normalized/standardized, describe the approach(es): specify linear or non-linear and define image types used for transformation OR indicate that data were not normalized and explain rationale for lack of normalization.*
Normalization template	*Describe the template used for normalization/transformation, specifying subject space or group standardized space (e.g. original Talairach, MNI305, ICBM152) OR indicate that the data were not normalized.*
Noise and artifact removal	*Describe your procedure(s) for artifact and structured noise removal, specifying motion parameters, tissue signals and physiological signals (heart rate, respiration).*
Volume censoring	*Define your software and/or method and criteria for volume censoring, and state the extent of such censoring.*

Statistical modeling & inference

Model type and settings	*Specify type (mass univariate, multivariate, RSA, predictive, etc.) and describe essential details of the model at the first and second levels (e.g. fixed, random or mixed effects; drift or auto-correlation).*
Effect(s) tested	*Define precise effect in terms of the task or stimulus conditions instead of psychological concepts and indicate whether ANOVA or factorial designs were used.*

Specify type of analysis: ☐ Whole brain ☐ ROI-based ☐ Both

Statistic type for inference<br>(See Eklund et al. 2016)	*Specify voxel-wise or cluster-wise and report all relevant parameters for cluster-wise methods.*
Correction	*Describe the type of correction and how it is obtained for multiple comparisons (e.g. FWE, FDR, permutation or Monte Carlo).*

Models & analysis

n/a	Involved in the study
☐	☐ Functional and/or effective connectivity
☐	☐ Graph analysis
☐	☐ Multivariate modeling or predictive analysis

Functional and/or effective connectivity	*Report the measures of dependence used and the model details (e.g. Pearson correlation, partial correlation, mutual information).*
Graph analysis	*Report the dependent variable and connectivity measure, specifying weighted graph or binarized graph, subject- or group-level, and the global and/or node summaries used (e.g. clustering coefficient, efficiency, etc.).*
Multivariate modeling and predictive analysis	*Specify independent variables, features extraction and dimension reduction, model, training and evaluation metrics.*
